# Effect of first dorsal interosseous strengthening on clinical outcomes in patients with thumb osteoarthritis: a study protocol for a randomized controlled clinical trial

**DOI:** 10.1186/s13063-022-06120-3

**Published:** 2022-03-03

**Authors:** Natália Barbosa Tossini, Natália Duarte Pereira, Gabriela Sardeli de Oliveira, Paula Regina Mendes da Silva Serrão

**Affiliations:** Physical Therapy Department, Federal University of Sao Carlos (UFSCar), Rodovia Washington Luis Km 235, São Carlos, São Paulo, CEP 13565-905 Brazil

**Keywords:** Osteoarthritis, Thumb, Exercise, Therapy, First dorsal interosseous

## Abstract

**Background:**

Thumb carpometacarpal osteoarthritis (CMC OA) is characterized by chronic progressive degeneration of the joint cartilage, with high prevalence. Patients present with pain at the base of the thumb, morning stiffness, and muscle weakness, symptoms that affect hand function and therefore interfere in activities and social participation. Movements that involve grip or lateral pinch are the most affected and directly impact independence, self-care, and leisure activities. In the literature consulted, several protocols with exercises for these patients were found. However, most do not compare the same intervention modality and only provide basic methodological information, with no consistent information on training load and load progression. In addition, most protocols only address the strengthening of the abductor and extensor thumb muscles and pinching or grasping exercises. However, some biomechanical and electromyographic studies have demonstrated the important role of the first dorsal interosseous muscles as stabilizers of the thumb carpometacarpal joint.

**Methods:**

This is a randomized, controlled, double-blind, and parallel clinical trial that will include 56 participants, over 40 years old, with radiographic evidence of thumb base osteoarthritis. Participants will be randomly allocated into two groups: control and intervention. The following evaluations will be conducted: the Australian/Canadian Hand Osteoarthritis Index, Canadian Occupational Performance Measure, Nine-Hole Peg Test, grip and pinch strength associated with muscle activation assessment, and Bilateral Upper Limb Function Test at four different times: baseline, session 13, session 18, and follow-up. Treatment will take place over 6 weeks, with reassessments in the fourth and sixth weeks and 3 months after the end of the intervention (follow-up). Qualitative variables will be expressed as frequency and percentage, and quantitative variables as mean and standard deviation. Intergroup comparison of the intervention will be performed by repeated measures ANOVA, considering the effect of the two groups and four assessments, and interactions between them.

**Discussion:**

This study will demonstrate whether the specific strengthening of the first dorsal interosseous muscle has a superior and positive effect on the clinical picture of patients with CMC OA. Additionally, if specific strengthening of the muscle is not superior to the traditional protocol in the literature, it will also be determined whether the two protocols are equivalent in terms of the best clinical picture.

**Trial registration:**

Brazilian Registry of Clinical Trials (ReBEC) RBR-8kgqk4. Prospectively registered on 15 January 2020

## Administrative information

**Table:** 

Title {1}	Effect of first dorsal interosseus strengthening on clinical outcomes in patients with thumb osteoarthritis: a study protocol for a randomized controlled clinical trial
Trial registration {2a and 2b}.	Brazilian Registry of Clinical Trials (ReBEC) ID: RBR-8kgqk4, date of registration: 15 January 2020.
Protocol version {3}	Version 1
Funding {4}	Not applicable
Author details {5a}	^1^ Physical Therapy Department, Federal University of Sao Carlos (UFSCar), Rodovia Washington Luis Km 235, São Carlos, São Paulo CEP 13565-905, Brazil.
Name and contact information for the trial sponsor {5b}	Name: Paula Regina Mendes da Silva Serrão. E-mail address: paula.serrao@ufscar.br Department of Physical Therapy, Federal University of São Carlos, Rodovia Washington Luiz, Km 235, São Carlos, São Paulo CEP 13565-905, Brazil.
Role of sponsor {5c}	Not applicable

## Introduction

### Background and rationale {6a}

Thumb carpometacarpal (CMC) osteoarthritis (OA) is a health condition characterized by progressive degeneration of the articular cartilage, subchondral bone sclerosis, ligament laxity, and osteophyte formation at the base of the first metacarpal [1]. It is a chronic and highly prevalent disease, especially in postmenopausal women. The Framingham Study found that 2% of men and 7% of women aged 40 to 84 years had symptomatic CMC OA, while radiographic CMC OA was present in 30% of men and 33% of women in the same study [2]. Another investigation estimated the prevalence of CMC OA to be 13% in people aged 41–50 years, increasing to 68% in 71- to 80-year-olds [3].

Patients with CMC OA have a lower pain threshold, pain at the base of the thumb or thenar eminence, morning stiffness, and muscle weakness, especially in the abductor pollicis and extensor muscles of the index finger [4]. These symptoms affect hand function, thereby interfering in the activities and social participation of patients [5]. The disease causes significant social and personal restrictions, reduced quality of life [6], and incapacity for work [7]. When compared to OA in the other fingers, patients with CMC OA receive more anti-inflammatory drugs and have a worse prognosis because they experience more severe pain and greater physical dysfunction [7]. Movements involving grip or lateral pinch are the most affected and directly impact independence, self-care, and leisure activities [8]. Activities reported as the most difficult to perform are opening jars, writing, turning keys in locks, opening food packages, wringing clothes, and carrying heavy objects between the thumb and fingers [9, 10].

The European League Against Rheumatism (EULAR) (2018) recommends that surgery for patients with CMC OA only be considered in the event of important structural abnormalities and when conservative treatment has not been effective in reducing pain and improving dysfunction [11–13]. In the early stages of the disease, conservative treatment has a beneficial effect on symptom relief [12] and includes patient education techniques, use of auxiliary devices, orthoses, and exercises [11]. However, the latest systematic review and meta-analysis on physical therapies (orthosis, mobilizations, neurodynamic techniques, and physical exercises) for patients with CMC OA highlighted that, despite the evidence on the unimodal or multimodal use of these treatments, only 5 studies were identified, suggesting that new high-quality randomized clinical trials are needed [14].

Although there are several exercise protocols for patients with CMC OA in the literature, most do not compare the same intervention modality [4, 15–18]. Additionally, the protocols only present basic methodological information, such as how many times a week the exercises should be performed and the number of repetitions for each exercise. However, there is no consistent information about training load and load progression methods. The majority of the protocols used the number of repetitions based on the weeks of treatment as an exercise progression method, that is, progression is general and not tailored to the patient’s capacity [4, 16, 17]. This contrasts with EULAR recommendations, which stipulate that OA management should be individualized [11].

The exercises proposed in the protocols describe the active movement of the CMC, proximal (PIP) and distal interphalangeal (DIP) joints of the thumb and other fingers, strengthening of the thumb abductor and extensor muscles, and pinching or gripping exercises [15, 17–19]. However, ligament laxity and joint hypermobility are known to be important etiological factors for the development of the disease [20, 21]. This laxity favors joint subluxation, which results in incongruity and abnormal joint loading, which may contribute to the development of CMC OA [22, 23]. In this respect, biomechanics and electromyography studies have demonstrated the important role of the opposing thumb and first dorsal interosseous muscle (1st DI) as stabilizers of this joint [24, 25].

The action of the 1st DI is described as antagonistic to the subluxation forces of the adductor pollicis muscle, that is, it centers the base of the 1st metacarpal on the trapezius [24–26]. As such, including the 1st DI muscle in CMC OA protocols seems to have important clinical significance. However, few protocols currently include exercises for this muscle [15, 27, 28], and there are no studies comparing two equal modalities in order to assess the effect of strengthening this muscle or not.

Thus, it is essential to carry out a study that adds to the physical exercise protocol the strengthening of the 1st DI muscles, in order to verify the effect of this strengthening on the clinical condition of patients with OA CMC. Therefore, it seems relevant, scientifically and clinically, to compare two protocols that use the same treatment technique (physical exercises) with the addition of the strengthening of the 1st DI. Furthermore, it is extremely important that the protocols clearly present the load used and the load progression parameters, as these variables can influence the result of the treatment.

### Objectives {7}

The main objective of this randomized clinical trial will be to verify whether a protocol with load increment, focusing on strengthening the 1st dorsal interosseous, will be effective for pain relief and improvement of strength and function for patients with OA CMC.

The secondary objectives will be to compare the clinical characteristics of the patients evaluated in the study, between the two treatment groups: such as pain at rest, pain during pinch movement and during activities, activity and social participation, manual dexterity, hand function, hand strength, grip and pinch, and muscle activation.

### Trial design {8}

**Fig. 1:**
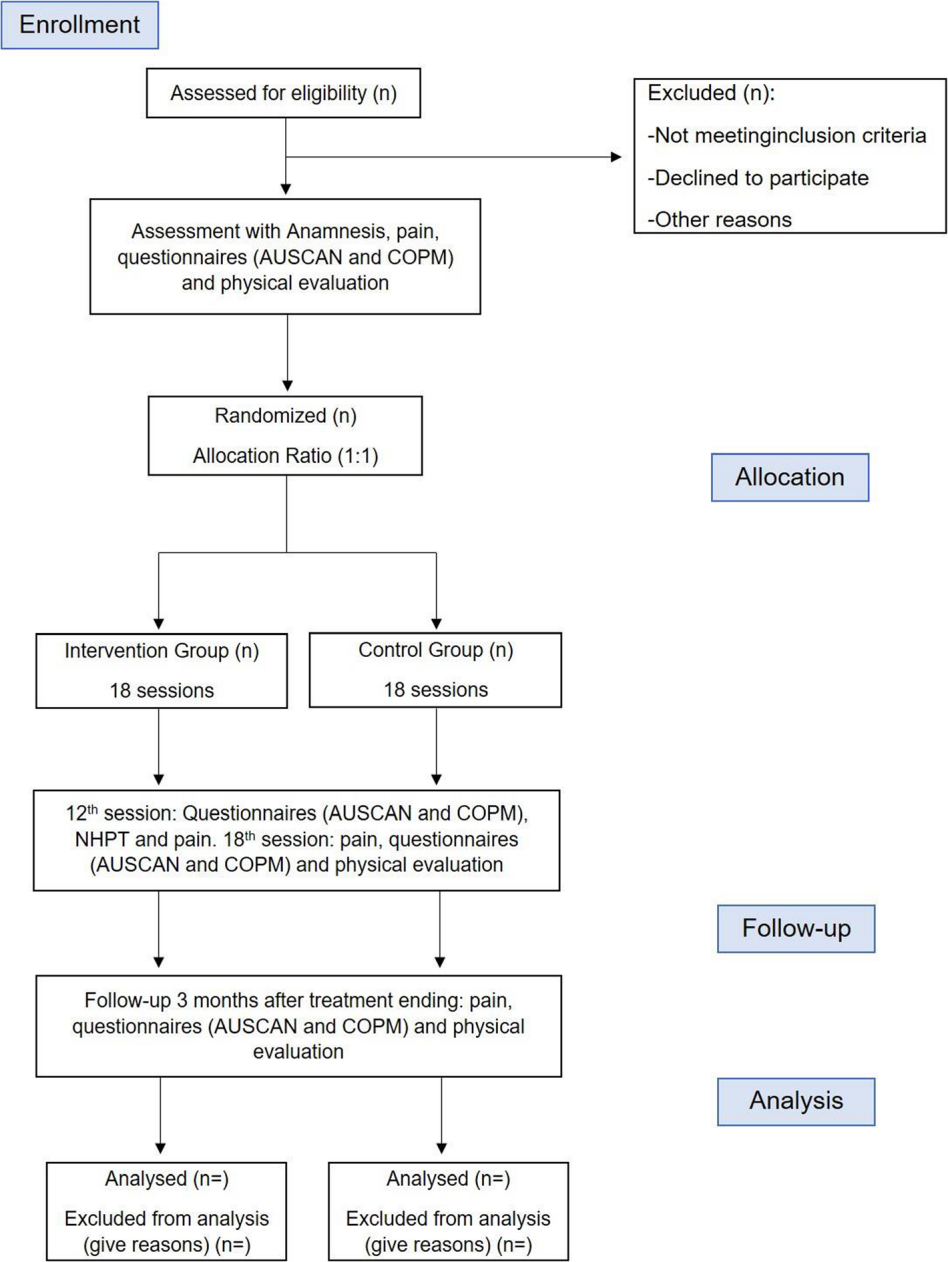
Study design flowchart

This is a randomized, controlled, double-blind (evaluator and patient), and parallel clinical trial. There will be 6 weeks of treatment, with reassessments in the fourth and sixth weeks and 3 months after the end of the intervention (follow-up), as shown in the study design flowchart (Fig. 1). The planning of this project followed the guidelines of the Consolidated Standards of Reporting Trials (CONSORT) and Standard Protocol Items: Recommendations for Interventional Trials (SPIRIT) guidelines.

## Methods: participants, interventions, and outcomes

### Study setting {9}

The trial is underway in the Research Laboratory in Rheumatology and Hand Rehabilitation, in the Physiotherapy Department of the Federal University of São Carlos (UFSCar) in São Carlos, Brazil.

Participants from the community of São Carlos, Brazil, will be invited to participate in the study. Participants will be recruited from social media, local and regional orthopedic and rheumatology clinics, newspaper and magazine advertisements, and leaflets posted on bulletin boards. After declaring an interest, participants will be phone interviewed by the principal investigator to see if they meet any non-inclusion criteria. If they are considered eligible, a face-to-face evaluation will be scheduled to confirm the inclusion criteria for the study.

### Eligibility criteria {10}

#### Inclusion criteria

Participants will be eligible for the study if they meet all the following inclusion criteria [17]: age over 40 years, radiographic evidence of thumb base OA read by a trained rheumatologist (Eaton-Littler-Burton criteria), normal score in the Mini-Mental State Examination (MMSE) according to schooling level [29], and average pain ≥ 30 on a 100-mm visual analog scale (VAS), where 0 is no pain and 100 the worst pain imaginable, over the past 30 days and in the 48 h prior to baseline assessment. In cases of bilateral OA TMC, the most severely affected hand will be included (according to VAS scores). If the pain scores are the same for both hands, participants will be asked to designate the worst hand (i.e., that causes the most problems, either dominant or non-dominant).

#### Non-inclusion criteria

Participants who meet any of the following criteria will not be included: known diagnosis of crystal arthritis, autoimmune arthritis, hemochromatosis, or fibromyalgia; hand surgery in the past 6 months or scheduled in the next 6 months; intra-articular hyaluronic acid injection in the affected joint in the past 6 months; intra-articular steroid injection in the affected joint in the past month; significant injury to the affected joint in the past 6 months; any other self-reported hand condition that likely contributes to pain at the base of the thumb; poor general health likely to interfere with compliance or assessments, judged by the investigator; women who are pregnant or breastfeeding; or current use of any of the study interventions.

#### Exclusion criteria

Participants who undergo intra-articular injection of steroids or hyaluronic acid, begin physical or occupational therapy for thumb base OA, and suffer upper limb trauma or fracture requiring surgical intervention or not during the proposed 6-week treatment period will be excluded from the study.

### Who will provide informed consent? {26a}

All eligible participants will receive information about the study, and the blinded evaluator will obtain written informed consent (IC) from participants prior to assessment.

### Additional consent provisions for collection and use of participant data and biological specimens {26b}

Not applicable

### Interventions

#### Explanation for the choice of comparators {6b}

The intervention group will perform manual thumb joint mobility and 1st DI strengthening exercises. The control group will be submitted to a standard manual exercise protocol with proven benefits, previously published in the literature. All the sessions for both groups will be carried out in person, on non-consecutive days, with one of the two physiotherapists trained to apply the protocols.

The intervention will take place over a 6-week period, with three 45-min sessions a week, totaling 18 treatment sessions. The first session will take place 1 week after the baseline assessment. Both intervention protocols will be applied based on the shaping method for exercise load progression, as described below.

#### Intervention description {11a}

##### Control group

The control group will perform a manual exercise protocol with traditional exercises for CMC OA patients, based on the protocol of Gravas et al [18]. The hand mobility and stretching exercises will be executed in the order described in the protocol, with 10 repetitions each, throughout the treatment period.

The strength and joint stability exercise will also be performed as described, but the volume and load progression will be different. Progression parameters will also be used for the load. As such, for exercises 4, 5, and 6:

- Volume: 10 repetitions of 15 s each
- Feedback measurement parameter (FMP): number of repetitions performed in 15 s
- Progression parameter 1 (PP1): increase the resistance of the elastic (different color elastic) or the mass

It is important to emphasize that exercise 5 will be performed with a silicone grip ball in order to ensure optimal load progression.

The exercises will be applied in the order described in the original protocol.

##### Intervention group

The group focused on strengthening the 1st DI will perform 6 mobility and 6 strengthening exercises, divided into even and odd treatment days. The mobility exercises will be interspersed with the strengthening exercises to prevent muscle fatigue.

The following are the even treatment session:

1. Thumb opposition: touch the tip of each fingertip with the thumb.

- Volume: 10 repetitions

2. Index finger abduction: palm and forearm flat on the table. The elastic will be placed around the PIP and fixed next to the 5th metacarpal. Perform index finger abduction by sliding it along the table.

- Volume: 10 repetitions of 15 s each
- Feedback measurement parameter (FMP): number of repetitions performed in 15 s
- Progression parameter 1 (PP1): increase the fixing distance by 2 cm
- Progression parameter 2 (PP2): increase the resistance of the elastic (different color)

3. Thumb abduction: start with the thumb lying flat against the palm, in line with the index finger. Move the thumb as far from the palm as possible, staying in line with the index finger.

- Volume: 10 repetitions

4. Dragging Styrofoam balls: palm on the board and forearm on the table. The Styrofoam ball with Velcro should be positioned on the middle phalanx of the index finger. Perform index finger abduction to push the ball over the board, without lifting the finger off the table.

- Volume: 10 repetitions
- Feedback measurement parameter (FMP): time taken (in seconds) to carry the ball from one end of the board to the other
- Progression parameter 1 (PP1): tilt the board
- Progression parameter 2 (PP2): place an elastic band around/on the PIP

5. Thumb CMC extension: move the thumb as far as possible from the palm, without hyperextending the thumb MP joint.

- Volume: 10 repetitions

6. Adduction and abduction of the index finger and thumb: the index finger and thumb semi-flexed and other fingers flexed. Perform adduction and abduction of the index finger and thumb to pick up wooden stars from within a 15-cm area.

- Volume: 10 repetitions
- Feedback measurement parameter (FMP): time taken to pick up stars in a 15-cm area
- Progression parameter 1 (PP1): increase the area to 30 cm
- Progression parameter 2 (PP2): increase the area to 45 cm

The following are the odd treatment session:

1. Thumb flexion: start with the thumb extended outward as far as possible from the palm. Flex the tip of the thumb to touch the base of the little finger.

- Volume: repeat 10 times

2. Index finger abduction: start the exercise with the drawer positioned in the center of the patient, 0 cm from the edge of the table. Open the drawers from the bottom to top and right to left using the side edge of the index finger.

- Volume: 10 repetitions of 30 s each
- Feedback measurement parameter (FMP): number of drawers opened in 30 s
- Progression parameter 1 (PP1): add elastic load behind each drawer
- Progression parameter 2 (PP2): increase the distance of all the elastics by 2 cm

3. Thumb IP flexion: bend only the tip of the thumb (IP joint).

- Volume: 10 repetitions

4. Aquarium net: start the exercise with the forearm in a neutral position supported on the backrest of the chair. The aquarium net will be placed in the patient’s hand, between the index finger and thumb. Perform abduction of the index finger, touching the lower edge of the table with the aquarium net.

- Volume: 10 repetitions
- Feedback measurement parameter (FMP): time taken (in seconds) to touch the bottom edge of the table with the aquarium net 10 times
- Progression parameter 1 (PP1): add 2 artificial ice cubes to the aquarium net

5. Thumb MP flexion: bend only the MP joint.

- Volume: repeat 10 times.

#### Criteria for discontinuing or modifying the allocated interventions {11b}

Participants are free to withdraw from the study at any time and for any reason without penalty. Participants will be withdrawn from the study if they begin a different treatment in another location.

#### Strategies to improve adherence to interventions {11c}

In order to avoid and minimize missing data, all participants will receive paper reminders with the date and time of each session. The investigator will also contact them by phone to reinforce this schedule. In addition, patients will be asked to inform the physiotherapist responsible for the treatment of their pain level that week (according to the VAS) of any complications on a weekly basis.

#### Relevant concomitant care permitted or prohibited during the trial {11d}

During the intervention and follow-up, participants will be given a diary to document on which days of the week and for how many hours they used orthoses. They will also be advised not to seek any treatment after the end of the intervention, and should they do so, it must be reported in writing. No guidance or restrictions will be given on the use of analgesic drugs, but participants will be asked to record the name of the drug, as well as the date and dosage used.

#### Provisions for post-trial care {30}

All study patients will be entitled to follow-up treatment if pain worsens after the intervention. Any harm suffered by patients due to participation in the study will be remedied. For ethical reasons, the treatment that produces the best results will be offered to the other group at the end of the study.

### Outcomes {12}

#### Primary outcomes

The primary outcomes will be change in pain scores at the base of the thumb, assessed by VAS (0–100 mm), at rest and during pinch movement from baseline to 6 weeks [30]; change in hand function, evaluated using the Australian/Canadian Hand Osteoarthritis Index (AUSCAN) (0–36) from baseline to 6 weeks [30]; change in values of pinch strength (Lafayette Hydraulic Pinch Gauge (in kg)) from baseline to 6 weeks; and change in magnitude of activation of the abductor pollicis brevis (APB), 1st DI, extensor pollicis brevis (EPB), and abductor pollicis longus (APL) from baseline to 6 weeks.

#### Secondary outcomes

The secondary outcomes will be change in VAS pain scores (0–100 mm) at the base of the thumb, at rest and during pinch movement from baseline to 4 and 18 weeks; change in hand function, assessed by the AUSCAN function section (0–36) from baseline to 4 and 18 weeks; change in social participation according to the Canadian Occupational Performance Measure (COPM) tool from baseline to 4, 6, and 18 weeks, change in pain based on the AUSCAN pain section (0–20) from baseline to 4, 6, and 18 weeks; change in manual dexterity as per the Nine-Hole Peg Test (NHPT) (expressed in seconds) from baseline to 4, 6, and 18 weeks; change in values of grip strength from baseline to 4, 6, and 18 weeks (Lafayette Professional Hand Dynamometer (in kg)) and pinch strength (Lafayette Hydraulic Pinch Gauge (in kg)) from baseline to 4 and 18 weeks; change in execution time (expressed in seconds) of Bilateral Upper Limb Function Test (BULFT) tasks from baseline to 4, 6, and 18 weeks; and magnitude of activation of the abductor pollicis brevis (APB), 1st DI, extensor pollicis brevis (EPB), and abductor pollicis longus (APL) from baseline to 4 and 18 weeks.

### Participant timeline {13}

Table 1 shows which evaluations will be performed on patients throughout the study period, i.e., in pre-treatment, during each session, and at follow-up.

**Table 1:** Participant timeline

	Pretreatment	1st	2nd	3rd	4th	5th	6th	7th	8th	9th	10th	12th	13th	14th	15th	16th	17th	18th	Follow-up
Evaluation form	X																	X	X
Pain	X												X					X	X
AUSCAN	X												X					X	X
COPM	X												X					X	X
NHPT	X												X					X	X
BULFT	X																	X	X
Grip strength	X																	X	X
Pinch strength	X																	X	X
Muscle activation	X																	X	X

*AUSCAN* Australian/Canadian Hand Osteoarthritis Index, *COPM* Canadian Occupational Performance Measure, *NHPT* Nine-Hole Peg Test, *BULFT* Bilateral Upper Limb Function Test

### Sample size {14}

A priori sample calculation was performed in the Gpower 3.1.5 software, considering the comparison of two independent groups (control and intervention) in four distinct stages (pretreatment, 13th and 18th assessment, and follow-up). The statistical test that will be applied was also taken into account, that is, repeated measures analysis of variance (ANOVA) for the variables of interest (pain, manual dexterity, function, strength, and muscle activation). It was considered an effect size of 0.25, 5% significance, and power of 90%, resulting in 46 participants (23 per group). Considering possible sample losses, we will evaluate 20% more, that is, 56 subjects divided into two groups of 28 each.

### Recruitment {15}

Participants will be recruited from social media, local and regional orthopedic and rheumatology clinics, newspaper and magazine advertisements, and leaflets posted on bulletin boards and dissemination in the university’s internal media.

### Intervention assignment: allocation

#### Sequence generation {16a}

Individuals who agree to take part in the study and meet all the study criteria will be assigned to the intervention or control group by 1:1 allocation, according to computer-generated randomization, using block sizes of 4 and 6. The two physiotherapists who will apply the interventions will also be randomized in order to avoid bias in blinding.

#### Concealment mechanism {16b}

The allocation sequence will be concealed from the researcher assessing participants in sequentially numbered opaque, sealed, and stapled envelopes. The envelopes will be kept in a locked drawer.

#### Implementation {16c}

All patients who give consent for participation and who fulfill the inclusion criteria will be assigned to the intervention group or control group through a 1: 1 allocation, according to computer-generated randomization, using blocks of sizes 4 and 6. A Master’s student not involved in participant assessment will prepare the sequence generation and envelopes. The study coordinator will open the envelopes only after the enrolled participant completes all baseline assessments. The estimated time between baseline assessments and allocation will not exceed 5 days. The envelopes will identify the physiotherapist to perform the intervention and which intervention the participant has been assigned to.

### Assignment of interventions: blinding

#### Who will be blinded {17a}

All clinical assessments will be conducted by an evaluator blinded to treatment allocation. In order to reduce blindness bias, participants will be instructed not to disclose any information about the treatment received during reassessment. In the event of unblinding of the assessor, its occurrence and the reason for it will be recorded and reported along with the trial results.

The two physical therapists who will coordinate the treatments will not be blinded to the group allocation, since both professionals will implement the two protocols. The statistician involved in the analysis will be blinded to the group allocation.

Participants will be blinded to the study hypothesis and their group allocation, but rather advised of the overall aspects involved in the treatment of both groups. They will not be informed of the specific treatments applied to each group or of the intergroup differences.

#### Procedure for unblinding if needed {17b}

Not applicable

### Data collection and management {18a}

#### Baseline assessment

This will consist of an evaluation form, questionnaires, and physical assessment. The assessments are summarized in Table 2 and separated according to the domains of the International Classification of Functioning, Disability and Health (ICF).

**Table 2:** Assessment according to CIF domains

Functionality and disability	
Body structures and functions	Activity and participation
Pain	AUSCAN
Grip strength	COPM
Pinch strength	NHPT
Muscle activation	BULFT

The evaluation form will characterize the participants’ personal information, such as age, sex, schooling level, occupation, dominant upper limb, anthropometric data (weight, height, body mass index), and demographic data. This form will also include questions related to the clinical history of the participants (e.g., time since diagnosis (in years), use of continuous medications, and orthosis). Participants will also be asked to report their pain level at four different times: average pain over the past 30 days, average pain in the 48 h prior to baseline assessment, and pain at the moment and during the execution of the lateral pinch.

##### Questionnaires

All participants will complete the AUSCAN, a self-reported questionnaire, specific to hand OA, and valid for assessing pain and/or disability in patients with CMC OA [31, 32].

The COPM, used to assess social participation, is characterized as an individualized measure since each participant scores which activities, he/she finds most difficult to perform. The tool encompasses three areas of occupational performance: self-care, productive, and leisure activities. Participants will rate the activities in order of importance on a scale of 1–10, with 1 being minor and 10 very important. Next, the five main occupational performance problems will be scored again, on a scale of 1–10, indicating patient performance in the task and their degree of satisfaction with their execution [33, 34].

##### Physical assessment

All participants will be assessed for manual dexterity, hand function, grip and pinch strength, and muscle activation.

Manual dexterity will be evaluated using the NHPT, a wooden board with nine holes (3 rows × 3 columns) that must be filled with nine wooden pegs. The participant will be instructed to place the pegs into the holes one at a time in any order and then remove them and place them in the container next to the board. The test will be timed [35] and performed bilaterally, starting with the dominant upper limb.

Hand function will be assessed using the activities in the BULFT, which involves performing thirteen tasks. The positioning of the participant and the evaluator’s instructions on executing each task will be in line with the test administration manual. All tasks performed will be timed.

Maximum grip and pinch (pulp-pulp, lateral, and tripod) strength will be determined with a manual hydraulic dynamometer (Lafayette Professional Hand Dynamometer) and Lafayette hydraulic pinch gauge, respectively. The recommendations of the American Society of Hand Therapists (ASHT) will be followed and participants will perform three repetitions of 6 s each, with 1-min rest intervals [36]. The average of the three repetitions will be used for statistical analysis. The tests will be performed bilaterally, starting with the dominant upper limb.

The magnitude of muscle activation will be measured during the grip and pinch strength tests, using a Trigno Wireless System (Delsys Inc., Boston, USA) at a sampling frequency of 1200 Hz and three Trigno mini wireless sensors (Trigno EMG sensor, Delsys Inc., Boston, MA), with a rejection rate higher than 80 dB. To evaluate the thumb APB, the mini electrode will be placed in the center of the thenar eminence, in the same direction as the thumb. For 1st DI assessment, the mini electrode will be placed on the dorsal surface of the hand, in the space between the index finger and thumb, while the participant performs the pinch movement. To evaluate EPB and APL, the mini electrode will be placed in the dorsal region of the forearm above the wrist and on the same side as the thumb, while the participant abducts the thumb [37]. Before positioning the electrodes, the skin in the area will be prepared as recommended by Surface Electromyography for the Non-Invasive Assessment of Muscles (SENIAM) [38].

#### Four-week assessment

After 4 weeks of intervention (13 sessions), a clinical reassessment will be performed by applying the AUSCAN questionnaire, the COPM tool, NHPT, and VAS for the average pain felt in the last 3 days, pain at that time, and pain during the execution of the lateral pinch movement.

#### Six-week assessment

At the end of the treatment (18 sessions), a second clinical reassessment will be conducted within 3 days of the last day of the intervention, repeating all the procedures described in the baseline assessment.

#### Eighteen-week assessment

The follow-up will be performed 18 weeks after the beginning of treatment, that is, 3 months after completion, when participants will undergo the same process described for baseline assessment.

### Plans to promote participant retention and complete follow-up {18b}

Participants will receive information about the study design and the importance of completing the final follow-up. The blind evaluator will contact the participants by telephone approximately 3 days before the scheduled date to arrange the best time and date for the final evaluation to be carried out. In addition, the physiotherapist who was responsible for administering the treatment will call the participants 15 days before the end date of the follow-up requesting them to report the mean pain over the last 15 days according to the VAS (0–100 mm).

### Data management {19}

The data are currently being collected in paper form and stored in binders in an allocated cabinet in the Physiotherapy Department of Federal University of São Carlos (UFSCar). After collection, all forms will be checked for data quality and missing information and then stored in a locked cabinet accessible only by the principal investigator. The data will be entered into the Excel 2011 software by the lead researcher. The database and electronic analyses will be stored on a secure computer server with personal login access authorized by the principal investigator, who will have access to the complete data set (blinded for group allocation), with the co-investigators granted access when necessary. After completion of the study, all data and study documents will be archived by the principal investigator and stored for 5 years in the Physiotherapy Department, in Federal University of São Carlos (UFSCar).

The data is not public and is in the possession of the main researcher, and if requested, it will be made available.

### Confidentiality {27}

The data will be treated anonymously and confidentially and at no time will the full name of the participants be disclosed at any stage of the study.

### Plans for collection, laboratory evaluation, and storage of biological specimens for genetic or molecular analysis in this trial/future use {33}

Not applicable.

## Statistical methods

### Statistical methods for primary and secondary outcomes {20a}

Data will be analyzed according to the intention-to-treat principle. All trial outcomes will be blinded for treatment allocation, and all participants will be analyzed in the treatment group to which they were originally randomized. Statistical analysis will be performed using the Statistical Package for the Social Sciences, version 19.0 (SPSS Inc., Chicago, IL, USA). A 5% significance level will be adopted for all analyses. The normality of the residues will be verified by the Shapiro-Wilk test. Pain, function, activity and participation, grip and pinch strength, dexterity, and muscle activation will be compared between the groups (control and intervention) and stages (pretreatment, 4- and 6-week assessments, follow-up), and interactions between them analyzed by mixed model ANOVA considering group and time factors for repeated measures if the residuals exhibit normality with post hoc contrasts (treated versus control group).

The first statistical procedure will be to verify, in general and at each assessment stage (pretreatment, 13th and 18th assessment, follow-up) if pain, function, activity and participation, grip and pinch strength, dexterity, and muscle activation differ between the intervention and control groups. The second statistical procedure aims to verify whether pain, function, activity and participation, grip and pinch strength, dexterity, and muscle activation change over the four assessment stages (pretreatment, 4- and 6-week assessments, follow-up) within each group.

The quantitative variables will be expressed as minimum and maximum values, means, medians, standard deviations, coefficients of variation, and asymmetry coefficients. Categorical variables will be expressed as frequencies and percentages.

### Interim analyses {21b}

Not applicable.

### Methods for additional analyses (e.g., subgroup analyses) {20b}

No additional analysis will be performed.

### Analysis methods to address protocol non-adherence and statistical methods to handle missing data {20c}

In cases of dropout or withdrawal from the study, the data will be assessed by intention-to-treat analysis. Participants that leave the study without performing the 18 proposed sessions (due to sickness, moving to another town, inability to attend sessions) will be considered protocol deviations.

### Plans to give access to the full protocol, participant-level data, and statistical code {31c}

Not applicable.

### Oversight and monitoring

#### Composition of the coordinating center and trial steering committee {5d}

A principal investigator (blind evaluator) and two coordinators who will coordinate all phases of the study and be responsible for statistical analysis and data interpretation will develop the study.

#### Composition of the data monitoring committee, its role, and reporting structure {21a}

There will be no data monitoring committee since only the primary evaluator and two study coordinators will have access to the clinical trial data.

#### Adverse event reporting and injuries {22}

If a participant withdraws from the trial, the reasons for withdrawal will be recorded, and all information provided up to the time of their withdrawal will be kept secret, maintaining data confidentiality. Strategies to maximize follow-up and prevent missing data will be used, including adhering to the assessment schedule in the event of participant withdrawal. Participants who withdraw from the trial will not be replaced.

All the medical records of volunteers will be carefully assessed, and any injuries or complications of the treatment, if any, will be reported along with the trial results. Injuries will be categorized as serious and minor adverse events.

#### Frequency and plans for auditing trial conduct {23}

Fortnightly meetings will be held with the group of researchers involved in the study in order to discuss the development of the study or other questions/doubts. An independent researcher will verify the data collected during the study. If any documents are missing or information is inconsistent, the local ethics committee will be notified. Finally, if there is any change in the study, the ethics committee, the journal, and the Brazilian Registry of Clinical Trials (ReBEC) will be notified immediately.

#### Plans for communicating important protocol amendments to relevant parties (e.g., trial participants, ethical committees) {25}

All amendments to the protocol will be communicated and approved by the Human Research Ethics Committee from the Federal University of São Carlos (UFSCar) – SP.

#### Dissemination plans {31a}

The results of this randomized controlled clinical trial are expected to be disseminated through presentations at national conferences and congresses and publication in peer-reviewed journals.

## Discussion

This study will demonstrate whether specific strengthening of the first dorsal interosseous muscle has a superior and positive effect on the clinical picture of patients with CMC OA. Additionally, if specific strengthening of this muscle is not superior to the traditional protocol in the literature, it will also be determined whether the two protocols are equivalent in terms of the best clinical picture. This will be analyzed based on possible changes in some symptoms (i.e., pain, function, activity, and participation) during the treatment period (4 weeks and 6 weeks), or 3 months after its completion.

To date, there are no guidelines for the treatment of CMC OA. The most recent update of treatment recommendations comes from the EULAR, which published an update on management recommendations for patients with hand osteoarthritis in 2018 [11]. However, the only recommendation for patients with OA at the base of the thumb is the use of orthosis for symptom relief. In general, the article indicates that the main goal of hand osteoarthritis treatment is to control symptoms such as pain and stiffness and optimize hand function in order to maximize activity, participation, and quality of life. Following this principle and developing a protocol involving muscle strengthening through functional activities in specific cases of CMC OA seems to be important. In addition, our protocol involves strengthening a muscle considered fundamental in stabilizing the joint involved, a significant differential.

Finally, it is worth mentioning that systematic reviews on the subject emphasize the low quality of clinical trials, which can interfere with the reliability and clinical applicability of manual exercises. As such, our protocol could contribute to the development of a reliable methodological procedure to guide physiotherapists in clinical practice, providing clear information on how to perform the exercises, training volume, and load progression.

## Trial status

The protocol registration was approved on March 11, 2020, and was registered on January 15, 2020. Recruitment was scheduled to begin on April 1, 2020, but has been postponed due to the COVID-19 pandemic.

## Data Availability

Not applicable.

## Abbreviations

OA: Osteoarthritis
CMC: Carpometacarpal
EULAR: European League Against Rheumatism
IFP: Proximal interphalangeal
1^st^ ID: 1st dorsal interosseous
UFSCar: Federal University of São Carlos
MMSE: Mini-Mental State Examination
VAS: Visual analog scale
ICT: Informed consent term
FMP: Feedback measurement parameter
pp1: Progression parameter 1
pp2: Progression parameter 2
AUSCAN: Australian/Canadian Hand Osteoarthritis Index
COPM: Canadian Occupational Performance Measure
NHPT: Nine-Hole Peg Test
BULFT: Bilateral Upper Limb Function Test
APB: Abductor pollicis brevis
EPB: Extensor pollicis brevis
APL: Abductor pollicis longus
ANOVA: Analysis of variance
CIF: Classification of Functioning, Disability and Health
ASHT: American Society of Hand Therapists
SENIAM: Surface Electromyography for the Non-Invasive Assessment of Muscles
ReBEC: Registro Brasileiro de Ensaios Clínicos
